# A descriptive survey of types, spread and characteristics of substance abuse treatment centers in Nigeria

**DOI:** 10.1186/1747-597X-6-25

**Published:** 2011-09-18

**Authors:** Peter O Onifade, Edward B Somoye, Olorunfemi O Ogunwobi, Adegboyega Ogunwale, Akinwande O Akinhanmi, Taiwo A Adamson

**Affiliations:** 1 Drug Addiction Treatment, Education and Research Unit, Neuropsychiatric Hospital, Aro, PMB 2002, Abeokuta, Ogun state, Nigeria

**Keywords:** Substance, abuse, treatment, Nigeria

## Abstract

**Background:**

Nigeria, the most populous country in Africa and the 8th most populous in the world with a population of over 154 million, does not have current data on substance abuse treatment demand and treatment facilities; however, the country has the highest one-year prevalence rate of Cannabis use (14.3%) in Africa and ranks third in Africa with respect to the one-year prevalence rate of cocaine (0.7%) and Opioids (0.7%) use. This study aimed to determine the types, spread and characteristics of the substance abuse treatment centers in Nigeria.

**Methods:**

The study was a cross sectional survey of substance abuse treatment centers in Nigeria. Thirty-one units were invited and participated in filling an online questionnaire, adapted from the European *Treatment Unit/Program Form (June 1997 version)*.

**Results:**

All the units completed the online questionnaire. A large proportion (48%) was located in the South-West geopolitical zone of the country. Most (58%) were run by Non-Governmental Organizations. Half of them performed internal or external evaluation of treatment process or outcome. There were a total of 1043 for all categories of paid and volunteer staff, with an average of 33 staff per unit. Most of the funding came from charitable donations (30%). No unit provided drug substitution/maintenance therapy. The units had a total residential capacity of 566 beds. New client admissions in the past one year totalled 765 (mean = 48, median = 26.5, min = 0, max = 147) and 2478 clients received services in the non-residential units in the past year. No unit provided syringe exchange services.

**Conclusions:**

The study revealed a dearth of substance abuse treatment units (and of funds for the available ones) in a country with a large population size and one of the highest prevalence rates of substance abuse in Africa. The available units were not networked and lacked a directory or an evaluation framework. To provide an environment for effective monitoring, funding and continuous quality improvement, the units need to be organized into a sustainable network.

## Background

Africa, with approximately one fifth of the world's population, is by far the continent with the least documentation in terms of data on substance abuse [1]. Nigeria, the most populous country in Africa and the 8th most populous in the world with an estimated population of over 154 million, does not have current data on substance abuse treatment demand and treatment facilities. The drug treatment demand figures quoted in the 2011 World Drug Report by UNODC [2] were sourced from 2004 data obtained from the government. The country has the highest one-year prevalence rate of Cannabis use (14.3%) in Africa and ranks 3rd in Africa with respect to the one-year prevalence rates of cocaine (0.7%) and opioids use (0.7%) [2]. Historically, the orthodox treatment of substance abuse in Nigeria took place in general psychiatric settings until 1983 when the first stand-alone substance abuse treatment unit was established [3]. Since then, many more substance abuse treatment units have evolved, existing alongside psychiatric units. But the current directory of the former is outmoded.

In 1998, because of the non-existence of a national database of existing structures and services developed to tackle the menace of drug abuse in Nigeria, the United Nations International Drug Control Program conducted a rapid situation assessment of drug abuse in Nigeria with one of the objectives being to determine "the availability, adequacy, nature and location of secondary and tertiary drug prevention services and personnel" [4]. The study was conducted in 22 of the 36 states, covering all the 6 geopolitical zones in the country. The study revealed that substance abuse treatment facilities existed in all the 22 states but largely as part of psychiatric, general or university teaching hospitals. The report also indicated the existence of traditional and religious centers for substance abuse treatment and rehabilitation. The assessment of these centers included only the names, location, number of personnel and facilities available.

Between 2002 and 2004, the Federal Ministry of Health and National Drug Law Enforcement Agency in collaboration with the United Nations Office on Drugs and Crime (UNODC), executed Project AD/NIR/02F22. The project had as part of its goals, the production of a directory of existing drug treatment and rehabilitation facilities in Nigeria and the conduct of a needs assessment of these centers [5,6]. In the 36 states of the country, the project identified forty-eight drug treatment centers, of which fourteen were selected for capacity building using 'stringent' criteria (not defined in the report). The directory produced by the project in 2004 contained the names and location of seventy-two treatment centers. It also contained basic information about some of the centers. However, the directory did not classify the centers into those designed primarily to treat substance abuse and those which treated substance abuse as a secondary problem in the context of psychiatric treatment. This study therefore aimed to determine the types, spread and characteristics of the treatment centers in Nigeria in order to address the aforementioned limitations of the earlier surveys and provide the much needed up-to-date data on substance abuse treatment capacity within the country.

## Methods

This was a cross sectional survey of the substance abuse treatment centers for the period between June 1, 2010 and May 31, 2011. Prior to this, we conducted a pilot survey involving 7 treatment units for the period between July 2008 and June 2009.

### Contact and recruitment process

Due to the absence of a recent directory for the pilot survey, we contacted the centers through a combination of e-mails, phone calls and snowballing technique. The Association of Psychiatrists in Nigeria provided a readily available pool of e-mail addresses of psychiatrists and allied professionals in Nigeria. The initial contacts yielded a list of 20 treatment centers, 7 of which participated in the pilot survey. For the main study, we recruited the centers during the UNODC-WHO organized Treatment Network (TREATNET II) training sessions which took place in various centers across Nigeria (Abeokuta, Kaduna, Calabar and Maiduguri) between June and December 2010 [7]. The sessions were attended by 374 individuals working in various treatment units in Nigeria.

### Instruments

For the pilot survey, we adapted the European *Treatment Unit/Program Form (June 1997 version) *[8,9], which had been used internationally to elicit information regarding substance abuse treatment units. The pilot survey informed further adaptation of the instrument for the main study. The instrument (see Additional file 1) was logically divided into three parts. The first part applied to all the invited units; the second part was applicable to only the units which provided residential services; while the third part was for low threshold/drop-in/outreach units. The measures in the instrument included contact information, basic unit characteristics, services, staffing, client characteristics, funding and evaluation.

We used LimeSurvey [10] to design the online form, which provided for necessary skips during questionnaire completion. LimeSurvey is a Free/Open Source software which allows users to quickly create intuitive and powerful, online question-and-answer surveys that can work for tens to thousands of participants without much effort. The survey software itself is self-guiding for participants. It has surpassed 400,000 downloads and is used by a significant number of large corporations, governmental institutions, academic facilities as well as private individuals around the world.

### Data collection procedure

A screening instrument (see Additional file 2) was first administered to determine the basic characteristics of the units represented at the TREATNET training sessions in Nigeria. Only the units which were explicitly set up as substance abuse treatment units were invited to participate in the main study. We designed an online version of the adapted *Treatment Unit/Program Form *and invited (via e-mail) the coordinator and/or the TREATNET contact in each eligible unit to participate in the survey between June 15 and 25, 2011. Access to the survey was restricted in such a way that no one could participate without invitation. We followed up the e-mail invitation with up to four e-mail reminders, phone calls and text messages. Partial and complete responses were saved in an online database which could be exported to Microsoft Excel, Comma Separated Value or Statistical Package for Social Sciences. The introduction page of the form contained a download link to the printable format of the form for respondents who preferred first filling the form offline. However, submission of responses was strictly online. Before data analysis, we reviewed each submission and verified unusual responses through phone calls.

### Data analysis

The online database was exported to Statistical Package for Social Sciences version 16 for descriptive analysis. Because of the outliers in the data, we used median and mode in addition to mean as measures of location [11].

## Results

### TREATNET II training participating units

Table 1 shows the basic information about the sixty-two treatment units which had at least one member of staff who participated in TREATNET training sessions in the country. Twenty-six (41.9%) belonged to the Federal Government; while 17 (27.7%) belonged to Non-Governmental Organizations (NGOs). Thirteen (21.0%) were outreach/preventive units, all of which were owned by NGOs; 24 (38.7%) were located in psychiatric or psychiatric departments of teaching and general hospitals. Only 5 (8.1%) of the units were stand-alone treatment centers. None of these stand-alone units belonged to the government. Only half of the units were officially dedicated to providing treatment of substance abuse, 17 (55%) of which belonged to NGOs, 10 (32%) to the Federal Government, 3 (10%) to private individuals and 1 (3%) to a State Government.

**Table 1:** TREATNET training participating substance abuse treatment units in Nigeria

Total = 62					
**Unit description**	**federal government**	**state government**	**private**	**NGO**	**Total**

**OFFICIALLY dedicated to substance abuse treatment**					
In the psychiatric department of a university teaching hospital	1 (1.6)	1 (1.6)	0 (0.0)	0 (0.0)	2 (3.2)
In a specialist psychiatric hospital	7(11.3)	0 (0.0)	1 (1.6)	0 (0.0)	8 (12.9)
Stand-alone drug dependence treatment unit (not part of a parent hospital)	0 (0.0)	0 (0.0)	1 (1.6)	4 (6.5)	5 (8.1)
In prison or any other law enforcement institution.	2 (3.2)	0 (0.0)	0 (0.0)	0 (0.0)	2 (3.2)
Outreach/preventive unit	0 (0.0)	0 (0.0)	0 (0.0)	13(21.0)	13(21.0)
Drug abuse research (excluding treatment) center	0 (0.0)	0 (0.0)	1 (1.6)	0 (0.0)	1 (1.6)
**Subtotal**	**10 (16.2)**	**1 (1.6)**	**3 (4.8)**	**17 (27.1)**	**31 (50.0)**
**Not OFFICIALLY dedicated to substance abuse treatment**					
In the psychiatric department of a UNIVERSITY TEACHING hospital	6 (9.7)	2 (3.2)	0 (0.0)	0 (0.0)	8 (12.9)
In the psychiatric department of a GENERAL hospital	1 (1.6)	3(4.8)	0 (0.0)	0 (0.0)	4 (6.5)
in a SPECIALIST PSYCHIATRIC hospital	1 (1.6)	1 (1.6)	0 (0.0)	0 (0.0)	2 (3.2)
In a general medical practice	2 (3.2)	1 (1.6)	1 (1.6)	0 (0.0)	4 (6.5)
In primary health care setting	1 (1.6)	0 (0.0)	0 (0.0)	0 (0.0)	1 (1.6)
in the administrative arm of government, e.g. ministry of health	0 (0.0)	1 (1.6)	0 (0.0)	0 (0.0)	1 (1.6)
In a counseling support unit for university students	2 (3.2)	2 (3.2)	2 (3.2)	0 (0.0)	6 (9.7)
University psychology department	2 (3.2)	1 (1.6)	0 (0.0)	0 (0.0)	3(4.8)
School of nursing/health technology	1 (1.6)	1 (1.6)	0 (0.0)	0 (0.0)	2 (3.2)
**Subtotal**	**16 (25.8)**	**12 (19.4)**	**1 (1.6)**	0 (0.0)	**31 (50.0)**

Grand total	26 (41.9)	13 (21.0)	6 (9.7)	17 (27.4)	62 (100)

### Needs survey participating units

All the 31 units invited to participate in the survey responded fully. As shown in Table 2, 15 (48.4%) and 1(3.2%) of the participants were located in the South-West and North-East geopolitical zones of the country respectively. NGOs owned most (58.1%) of them. A large proportion of the units (48.4%) owned the building space they occupied. With the exception of four, all the units with buildings of their own belonged to the federal government. More than half came into existence in the last decade. Only 5 (16.1%) of the units were described as treatment unit in a prison; 17 (54.8%) and 16 (51.6%) were specialized non-residential and specialized residential units respectively. Table 3 shows that the most common service provided by the units was short-term crisis or informal counseling support for people with substance abuse. This was provided by 25 (80.6%) of the units. Twenty-three (74.2%) reported significant change in the number of clients served in the past one year. About half of the units performed internal or external evaluation of treatment process or outcome but only 4 (12.9%) had available reports on the evaluation data.

**Table 2:** Characteristics of the substance abuse treatment units

Total = 31		
Variable	n	%
**Location: Geopolitical zone**		
North-Central	5	16.1
North-East	1	3.2
North-West	3	9.7
South-East	3	9.7
South-South	4	12.9
South-West	15	48.4
**Ownership**		
Federal Government	11	35.5
State Government	1	3.2
Non-Governmental Organization	18	58.1
Private	1	3.2
**Building space occupied exclusively by this treatment unit/Program in the fiscal year**		
No building space	2	6.5
Building space is shared with some other body free of charge	4	12.9
Rented	10	32.3
Owned	15	48.4
**The unit has a parent organization to which it belongs**		
Yes	16	51.6
**Year established**		
Before 1980	2	6.5
1981 - 1986	1	3.2
1986 - 1990	4	12.9
1991 - 1995	1	3.2
1996 - 2000	6	19.4
2001 - 2005	6	19.4
2006 - 2010	11	35.5
**Description**		
Specialized residential (e.g. therapeutic community, drug abuse unit standing alone or within a parent hospital)	16	51.6
Specialized non-residential (e.g. Low threshold/drop-in/street agency/outreach programs)	17	54.8
Non-Specialized residential (e.g. general hospital)	4	12.9
Non-Specialized non-residential (e.g. primary health care unit, general outpatient clinic, university counseling unit)	6	19.4
Treatment unit in Prison	5	16.1

**Table 3:** Services, changes and evaluation of the substance abuse treatment units

Total = 31		
Variable	n	%
**Services provided**		
Services aimed at detoxification and abstinence	21	67.7
Services aimed at drug-related harm reduction	24	77.4
Nonmedical and medical interventions services for drug abuse	22	71.0
Short-term crisis or informal counseling support for people with drug abuse	25	80.6
Structured longer-term programs for people with drug abuse	19	61.3
**Areas of significant changes in the units between June 1, 2010 and May 31, 2011**		
Treatment approach	22	71.0
Financial support	9	29.0
Staff composition	18	58.1
Client composition	21	67.7
Number of clients served	23	74.2
Staff-to-client ratio	14	45.2
**Evaluation**		
Participation in any internal or external evaluation of treatment process or outcomes	15	48.4
The treatment unit/Program itself conducts the evaluation	11	35.5
Government institution conducts the evaluation	1	3.2
University or research institution conducts the evaluation	2	6.5
Independent evaluation consultant conducts the evaluation	0	0.0
Others (funders, PhD psychology students) conduct the evaluation	2	6.5
Reports are available on the evaluation data	4	12.9

### Staffing

Most of the units (61.3%) did not employ people recovering from substance abuse. There were a total of 1043 people for all categories of paid and volunteer staff, giving an average of 33 staff per unit (median = 17, mode = 11, min = 3, max = 222). Seven hundred and eighty-three (75.1%) were paid (full/part time) staff. Table 4 shows that majority of full time paid staff were nurses with an average of 7 per unit (median = 2, min = 0, max = 45).

**Table 4:** Categories of staff of the substance abuse treatment units

Total = 31						
**Variable**	**Sum**	**Mean**	**Median**	**Mode**	**Min**	**Max**
**Staffing**						

Psychiatrists						
full time staff	38	1.23	0	0	0	8
part time staff	32	1.03	0	0	0	10
full time volunteers	6	0.19	0	0	0	2
part time volunteers	9	0.29	0	0	0	2
Other physicians						
full time staff	28	0.09	0	0	0	10
part time staff	18	0.58	0	0	0	4
full time volunteers	16	0.52	0	0	0	10
part time volunteers	18	0.58	0	0	0	4
Psychologists						
full time staff	50	1.61	1.0	0	0	8
part time staff	36	1.16	0	0	0	15
full time volunteers	16	0.52	0	0	0	8
part time volunteers	13	0.42	0	0	0	3
Social workers						
full time staff	59	1.90	1	0	0	8
part time staff	11	0.35	0	0	0	3
full time volunteers	13	0.42	0	0	0	10
part time volunteers	23	0.74	0	0	0	6
Nurses						
full time staff	221	7.13	2	0	0	45
part time staff	16	0.52	0	0	0	5
full time volunteers	3	0.1	0	0	0	1
part time volunteers	12	0.39	0	0	0	4
Informal counselors						
full time staff	37	1.19	0	0	0	10
part time staff	11	0.35	0	0	0	5
full time volunteers	11	0.35	0	0	0	8
part time volunteers	45	1.45	0	0	0	15
Other therapists (rehabilitation specialists, trainers, educators)						
full time staff	56	1.81	0	0	0	8
part time staff	31	1.00	0	0	0	11
full time volunteers	9	0.29	0	0	0	3
part time volunteers	23	0.74	0	0	0	6
Clerks, administrators, maintenance personnel						
full time staff	121	3.90	4	0	0	22
part time staff	18	0.58	0	0	0	10
full time volunteers	23	0.74	0	0	0	20
part time volunteers	20	0.65	0	0	0	15

### Funding

Practically no funds were received from health insurance (Table 5). Most of the funding came from charitable donations (29.9%). Fourteen percent came from clients' private income. Only 6 NGOs received funds (ranging between 40% and 100%) from international organizations.

**Table 5:** Proportion of funding from various sources

Total = 31					
**Source of financing**	**Percentage**				
	
	**Mean %**	**Median %**	**Mode %**	**Min %**	**Max %**

Federal Government	28.94	0	0	0	100
State Government	3.06	0	0	0	90
Local Government	0.16	0	0	0	5
International Organization	12.97	0	0	0	100
Client fees: private income	14.16	0	0	0	100
Client fees: public insurance	0.10	0	0	0	3
Client fees: private insurance	0.00	0	0	0	0
Interest on capital or investments by the unit	2.42	0	0	0	40
Donations (charitable)	29.87	10	0	0	100

### Residential units

The 16 residential units in this study provided no drug substitution/maintenance therapy (Table 6). Fourteen (87.5%) had planned duration of successful treatment of between 3 months and 2 years. Eleven (68.8%) developed written individual treatment plan for the clients. Intake assessment was conducted in 15 (93.8%) of the units but 9 (56.2%) either did not use Addiction Severity Index or used it only occasionally. Most of the units (68.8%) kept their data only on paper.

**Table 6:** Characteristics of only the Residential substance treatment units

Total = 16		
Variable	n	%
**Treatment modality**		
Long term drug substitution/maintenance	0	0.0
Medication free therapy/long term psychosocial treatment	12	75.0
Advice/counseling/support	15	93.8
**Typical planned duration for successful treatment for the majority of clients**		
less than 3 months	2	12.5
At least 3 months (and less than 6 months).	7	43.8
At least 6 months (and less than 1 year)	4	25.0
1 to 2 years	3	18.8
**Written information and treatment plan**		
Provision of written information to clients about the treatment/services offered	7	43.8
Written, individual treatment plan usually developed for the clients	11	68.8
Individual treatment plan is based on Addiction Severity Index (ASI)	9	56.2
**Common treatment plan in the unit**		
Informal treatment plan	6	37.5
Formal written treatment plan - not signed by the client	10	62.5
Formal written treatment plan - signed by the client	5	31.2
**Assessment and data management**		
Intake or initial assessment conducted	15	93.8
Occasional use of Addiction Severity Index (ASI) for intake or initial assessment purposes	5	31.2
Routine use of ASI for intake or initial assessment purposes	7	43.8
ASI not used for intake or initial assessment purposes	4	25.0
Data are kept on both paper and in the computer	5	31.2
Data are kept only on paper	11	68.8

The units' capacity, admissions and special populations between June 2010 and May 2011 are presented in Table 7. The total capacity was 566 with average of 35 clients per unit (median = 27, mode = 20, min = 12, max = 80). New client admissions totalled 765 (mean = 48, median = 26.5, min = 0, max = 147). In the past one year, there was average of 42.9% (median = 42.5%, min = 0%, max = 96%) dual diagnosed clients. The average rate of treatment completion was 70.2% (median = 79%, min = 0, max = 100).

**Table 7:** Residential substance abuse treatment units' capacity, admissions and special population

Total = 16						
**Variable**	**Sum**	**Mean**	**Median**	**Mode**	**Min**	**Max**

**Capacity and admission**						
Treatment unit's client capacity	566	35.38	27.5	20	12	80
Clients admitted in the unit, including readmissions, between June 1, 2010 and May 31, 2011	1132	70.75	38	20	0	266
Clients admitted more than once between June 1, 2010 and May 31, 2011.	155	9.69	3	0	0	65
New clients (Clients who had not asked for help in unit before) admitted in the unit June 1, 2010 and May 31, 2011.	765	47.81	26.5	0	0	147
**Special population on admission**						
Dual diagnosed currently on admission (%)		40.425	30	5.0	0	100
Dual diagnosed: past one year (%)		42.904	42.5	45	0	96
Adolescents: currently on admission(%)		17.2	10.5	0	0	88
Adolescents: past one year (%)		19.28	7.5	0	0	75
Probationers or parolees (%)		5.29	0	0	0	25
Probationers or parolees: past one year (%)		7.36	0	0	0	46
Females: currently on admission(%)		6.16	2	0	0	20
Females: past one year (%)		11.03	5	0	0	60
**Treatment completion June 1, 2010 and May 31, 2011**						
Completed (%)		70.17	79	60	0	100
Dropped out (%)		9.01	2.5	0.0	0	40
Absconded (%)		4.33	1.5	0	0	25
Premature discharge due to non-payment of overdue fees (%)		2.56	0	0	0	15
Premature discharge due to use of illicit drug within or outside the premises (%)		4.16	0	0	0	20
Premature discharge due to violent behavior (%)		2.96	1	0	0	20
Premature discharge due to violation of other rules and regulations of the unit (%)		1.56	0	0	0	10

All the units provided aftercare on-site (Table 8). Twelve (75.0%) and 10 (62.5%) provided primary and psychiatric care respectively. Six (37.5%) considered it the responsibility of the clients' relatives to provide housing for the clients.

**Table 8:** The accessible services in the residential substance abuse treatment units

Total = 16				
Service	Mostly the responsibility of the relatives	No	Yes, by referral	Yes, On-site
Primary medical care	0 (0)	2 (12.5)	2 (12.5)	12 (75.0)
Psychiatric care	0 (0)	2 (12.5)	4 (25.0)	10 (62.5)
Housing assistance	6 (37.5)	3 (18.8)	2 (12.5)	5 (31.2)
School or academic training	4 (25.0)	4 (25.0)	4 (25.0)	4 (25.0)
Vocational training	0(0)	6 (37.5)	3 (18.8)	7 (43.8)
Financial assistance	5 (31.2)	6 (37.5)	1 (6.2)	4 (25.0)
Job finding assistance	4 (25.0)	4 (25.0)	3 (18.8)	5 (31.2)
Aftercare	0 (0.0)	0 (0.0)	0 (0.0)	16 (100.0)

### Non-Residential units

The total number of clients who received services in the 15 non-residential units between June 2010 and May 2011 was 2478 (mean = 165, median = 20, min = 0, max = 2000). Table 9 shows that all the non-residential units provided outreach work and advice to drug users, but none provided syringe exchange services. Six (40.0%) provided written information to clients and 10 (66.7%) kept clients' records only on paper.

**Table 9:** Characteristics of only the Non-Residential substance treatment units

Total = 15		
Variable	n	%
**Accessible services in the units**		
Advice to drug users	15	100.0
Advice to Non-users	12	80.0
Advice to other service providers	10	66.7
Legal advice/assistance to users	6	40.0
Financial assistance to users	4	26.7
Housing for users	4	26.7
Education	13	86.7
Training	11	73.3
Job finding	5	33.3
Night shelter	1	6.7
Drop in sessions	7	46.7
Relapse Prevention	7	46.7
Prison work	5	33.3
Self help Group work	7	46.7
Counsellor-led group work	10	66.7
On site Syringe exchange	0	0.0
Mobile Syringe exchange	0	0.0
Outreach work	15	100.0
Drug Testing	2	13.3
Medical interventions - primary Health care	6	40.0
Alternative-complementary Therapies	8	53.3
**Brochure or other written material**		
Provision of written information to clients about the treatment/services offered (e.g., a brochure or other written material)	6	40.0
**Record management**		
Individual records on clients are available	14	93.3
**Are individual records identifiable**		
No, only frequency counts of contacts are kept	2	13.3
Not applicable	1	6.7
Yes, by anonymous identification (code)	4	26.7
Yes, by name of client	8	53.3
**Medium of record keeping**		
Records are kept on both paper and in the computer	4	26.7
Records are kept on paper	10	66.7
Not applicable	1	6.7

## Discussion

### Participating units

The study identified 31 units which were dedicated to the treatment of substance abuse in Nigeria. It was difficult to estimate the proportion of actual existing units in the country that this figure represented as there was no updated map or directory of substance abuse treatment units in the country. This untoward scenario would not have existed had there been a registration/accreditation institution for such units in the country. The ministry of health in each state of the country registers and accredits hospitals while the Corporate Affairs Commissions registers companies, associations and NGOs which might be parent bodies to some of these substance abuse treatment units. In other words, for now, registration of substance abuse treatment units in Nigeria is inferred from the registration of their parent bodies.

The last published directory of treatment centers compiled by UNODC Nigerian country office in 2004 through project AD/NIR/02/F22 contained 72 centers [6], many of which were psychiatric hospitals but not necessarily substance abuse treatment units. The (2004) project, using 'stringent' criteria (not defined in the report), identified only 14 of these units for capacity building. It is not impossible that there could be up to a hundred units currently actively providing substance abuse treatment services in the country. However, this projection contrasts sharply with the 15,213 similar units identified in the United States in 1999 [12], a country with only just about twice the population of Nigeria [13].

More than 90% of the units came into existence as from 1980's, in response to the sharp increase in heroin and cocaine use noted in that period [14]. The distribution of the units was skewed towards the South-West geopolitical zone with a share of 48.4%. This was similar to the picture in the 2004 directory [6] and might be responsible for the poorer access to treatment by people with substance abuse problems living in the Northern zones as reported by Adelekan & Lawal [14]. However the proportion in the North-Central rose from 9.7% in 2004 to 16.1% in this study. Adelekan & Lawal [14] also reported that traditional healing homes were a readily available form of treatment for substance abuse in the country; nonetheless, only about 3.3% to 24.8% of individuals, depending on the region, had access to any form of substance abuse treatment, the cost of treatment being the principal barrier to access.

Considering that Government was the main role player in the health care delivery in the country, the ownership of most of the units by Non-Governmental Organizations suggested that the community had responded more than the government to the problem of substance abuse. The significant changes in treatment approach reported by 71.0% of the units might be a result of the TREATNET capacity building training which took place between June and December 2010.

### Funding

There was inadequate funding of the NGO units as most of them had no buildings of their own and received no government or international funding. The National Health Insurance Scheme in Nigeria had minimal contribution to the treatment of people with substance abuse. The scheme [15] is a social security system which seeks to guarantee the provision of needed health services to persons on the payment of token contributions at regular intervals. However it makes provision for admission in a ward for only 15 days in a year and, for now, has well established program for only the formal sector, consisting of the public service and organized private sector; whereas two-third to three-quarter of those accessing substance abuse treatment in Nigeria are unemployed [16] and are thereby effectively barred from the funding opportunities afforded by the health insurance.

### Staffing

The predominance of nurses in the staffing, especially in the residential units, suggested a tendency towards the clinical model for the management of substance abuse, likely as a result of high proportion of dual diagnosed patients. The clinical setting would naturally be expected to be a conducive environment for long term substitution/maintenance therapy. On the contrary, none of the units embraced the therapy, despite the demand for treatment by persons who abused opiates [16]. Two possible reasons for this are, one, methadone and other substitution/maintenance medications are yet to be approved by the National Agency for Food and Drug Administration and Control; and two, at present, there is no existing framework for their use in the country. The staffing of 75.1% paid (full/part time) personnel suggested either poor volunteer recruitment efforts or the reluctance of the community towards rendering services without financial reward.

### Services and evaluation

The units provided a variety of services as a group. Most common were short-term crisis/informal advice and harm reduction services. The least common was structured longer-term services. Most residential units regarded the clients' relatives as being responsible for the housing, academic training, financial and job finding assistance. This is a great gap in meeting the clients' needs since the services left to the clients relatives are important components of comprehensive drug abuse treatment. Matching treatment services to an individual's particular problems is critical to his or her ultimate success in returning to productive functioning in the family, workplace, and society [17]. Though majority of the units reported providing harm reduction services, none provided syringe exchange despite the recent finding that injection drug users exist in all the regions of Nigeria with current prevalence rate of 8% among street drug users, who reported heroin, cocaine, speedball and pentazocine as the main substances injected [14].

Half of the units had no evaluation done on their treatment process or outcomes. Non-availability of evaluation data undermines any quality improvement effort in the units. Because there is still much to be learnt about the most effective, efficient and humane ways to help people with substance use disorders and to reduce the associated harm to users themselves and to others, it has been recommended that evaluation should be a feature of treatment systems and treatment policy to identify treatment needs, plan needs-based interventions, show if these interventions are consistent with needs and plans, and assess effectiveness and efficiency [18].

### Treatment capacity and demand

While the residential units had total capacity of 566 and rendered services to 1132 clients in the past one year, the non-residential units attended to 2478 clients. This was a remarkable increase compared with the 925 figure for 2009 [2]. But in a population of 154 million and prevalence rate of 0.7 for opiates and cocaine, these figures are grossly low. The high prevalence of dual diagnoses might be due to the location of the majority of residential units within psychiatric hospital premises [3].

### Limitations

This study may be unable to ascertain that all the available units actively providing substance abuse treatments were included or reached for the survey mainly as a result of the out-dated directory of such facilities within the country. The treatment units in psychiatric, general and teaching hospitals which were not "officially" designated as substance abuse units were excluded from this survey although some of their staff attended the TREATNET training on substance abuse treatment and therefore had obtained some level of competence to provide drug abuse treatment services. Also excluded were traditional healing homes which also provided substance abuse treatment. Thus, the current apparent capacity of the country in terms of substance abuse treatment facilities may somewhat fall short of actual reality.

### Strengths

This study is the first in Nigeria to conduct a comprehensive survey of substance abuse units using a standardized assessment instrument. The online platform used for the study has advantages of cost effectiveness and ease of data management.

## Conclusions

This study suggested a remarkable shortage of substance abuse treatment units for a country with a large population size and one of the highest prevalence rates of substance abuse in Africa. The available units were not organized into a network with an up-to-date directory. The observable but unimpressive improvement in treatment demand figures, the uneven distribution of treatment centers, the under-funding and inadequate government attention to the issue of substance abuse treatment are legitimate concerns raised by our findings.

In the light of the above, we recommend that the units be organized into a sustainable network with an up-to-date directory, a central annual evaluation of treatment process/outcomes and a central drug abuse information system. TREATNET is a pragmatic candidate in this regard and may serve as the template for a national framework. More and better funded units need to be set up by the public and private sectors, and Non-Governmental organizations. Advocacy for more volunteers to participate in the treatment provision is also recommended. A framework for the implementation of drug substitution/maintenance therapy should be designed. The over-arching importance of political will on the part of government in actualizing most of the identified strategies cannot be overemphasized.

## List of abbreviations

NGOs: Non-Governmental Organizations; TREATNET: Treatment Network; UNODC: United Nations Office on Drugs and Crime; WHO: World Health Organisation.

## Competing interests

The authors declare that they have no competing interests.

## Authors' contributions

POO and TAA conceptualized and designed the study. POO and AOA adapted the instrument for data collection. EBS, AO, and OO revised the adapted instrument. POO designed the online instrument and analyzed the data. All authors participated in contacting the treatment centers, drafting or revising the manuscript and giving approval for the version for publication.

## Authors' information

POO, TAA, AOA, EBS, and AO are consultant psychiatrists. OO is a psychiatric resident doctor.

## Supplementary Material

Additional file 1 **Substance abuse treatment unit questionnaire**. Printable version of the online form for collecting data on substance abuse treatment unit. Click here for file

Additional file 2 **Substance abuse treatment unit screening form**. The form distinguishes between generic treatment units and substance abuse treatment specific units. Click here for file
