# Supplementary material for: A descriptive survey of types, spread and characteristics of substance abuse treatment centers in Nigeria
Source: Subst Abuse Treat Prev Policy. 2011 Sep 18;6:25. doi: 10.1186/1747-597X-6-25 (PMC3182902; doi:10.1186/1747-597X-6-25)
Supplement: Additional file 1 — Substance abuse treatment unit questionnaire. Printable version of the online form for collecting data on substance abuse treatment unit. [file 1747-597X-6-25-S1.PDF]

Substance abuse treatment unit questionnaire

Needs assessment of all substance abuse treatment units in Nigeria

NB: ***This is the printable version of the online form. It is for preview ONLY so that you can have idea of all the questions before you start filling the online form. Questions 1 to 26 will be presented online for all treatment units. Question 27 to 37 will be presented only to centers which provide RESIDENTIAL treatment program. Your center will be regarded as RESIDENTIAL If for question 12, you select "Specialized Residential" as one of the options; otherwise the online form will regard your unit as non-residential. Questions 38 to 44 will be presented online to ONLY the unit which DO NOT provide RESIDENTIAL treatment program. The online form will automatically jump the questions which are not applicable to you.***

Dear colleague, thank you for participating in the TREATNET training in Nigeria. As you are aware, formation of the network of the drug dependence centers in Nigeria is one of the missions of TREATNET. We have some details about every centre which participated in the training. But we will like you to give us extra details about your unit so that we can determine the basic needs of each unit. Some units participated in this survey before the training. Now we want to include all the units as part of efforts towards forming and maintaining the network.

We notified you of this exercise because we have identified you as the head of your unit or the TREATNET representative in your unit.

We will be glad if you can complete filling this form by the 25th June.

For clarification, please contact Dr P.O Onifade 08035061082, onifadepo@neuroaro.com.

UNLESS OTHERWISE STATED, ALL QUESTIONS ARE BASED ON THE PERIOD BETWEEN JUNE 1 2010 AND MAY 31 2011.

There are 44 questions in this survey

Respondent's basic information

This section collects information on the contact who is filling this form

1 [r1]Your Surname *

Please write your answer here:

2 [r2]Your other names *

Please write your answer here:

3 [r3]Your phone number *

Please write your answer here:

4 [r4]What is your position in the unit/programme? *

Please write your answer here:

State the official post you hold in the unit

Basic information about the centre

This section is for the basic information about your centre

5 [b1]Name of treatment unit/programme *

Please write your answer here:

State the name of the unit as it is officially called

6 [b2] Street address where the unit is located *

Please write your answer here:

7 [b3]Town *

Please write your answer here:

8 [b4]State *

Please choose **only one** of the following:

- Abia
- Abuja Federal Capital Territory
- Adamawa
- Akwa Ibom
- Anambra
- Bauchi
- Bayelsa
- Benue

Borno
- Cross River
- Delta
- Ebonyi
- Edo
- Ekiti
- Enugu
- Gombe
- Imo
- Jigawa
- Kaduna
- Kano
- Katsina
- Kebbi
- Kogi
- Kwara
- Lagos
- Nasarawa
- Niger
- Ogun
- Ondo
- Osun
- Oyo
- Plateau
- Rivers
- Sokoto
- Taraba
- Yobe
- Zamfara

9 [b5]Phone number of the unit (if available)

Please write your answer here:

10 [b6]Email of treatment unit/programme (if available)

Please write your answer here:

Treatment unit characteristics

11 [c1]Does your unit/centre provide any of the following services (select all the apply)? *

Please choose **all** that apply:

- ☐ Services aimed at detoxification and abstinence
- ☐ Services aimed at drug-related harm reduction
- ☐ Nonmedical and medical interventions services for drug abuse
- ☐ Short-term crisis or informal advice, counselling support for people with drug abuse
- ☐ Structured longer-term programmes for people with drug abuse

12 [c2]Which of the following types of treatment programme correctly describes your treatment unit/programme? (INDICATE AS MANY AS ARE APPLICABLE TO YOUR UNIT) *

Please choose **all** that apply:

- ☐ Specialized Residential (eg therapeutic community, drug abuse unit standing alone or within a parent hospital)
- ☐ Specialized Non-Residential (eg Low threshold / drop-in / street agency / outreach programs)
- ☐ Non-Specialized Residential (eg general hospital)
- ☐ Non-Specialized Non-Residential (eg primary health care unit, general outpatient clinic, university counseling unit)
- ☐ Treatment Unit in Prison
- ☐ other (spsceify): ____________

NB: **Specialized** Drug abuse treatment unit is specifically designed for drug abuse clients who might or might not have associated health problems like HIV or psychiatric disorder. Any client without drug use problems is excluded from enrollment. Whereas, **Non-Specialized unit**, by default, provides general health services or non-addiction specialist services such as mental health, pain management and so on. It takes care of clients with drug use problems and those without them.

13 [c3]What is the ownership of your unit? *

Please choose **only one** of the following:

- ○ Federal Government
- ○ State Government
- ○ Local Government
- ○ Private
- ○ Non-Governmental Organization
- ○ Public-Private

14 [c4]Does the unit have a parent organization to which it belongs? *

Please choose **only one** of the following:

- ◯ Yes
- ◯ No

A parent organization may be a teaching hospital, a psychiatric hospital, a university etc in which the drug abuse treatment unit is an arm / department / unit

15 [c5]What is the name of the parent organization? *

Only answer this question if the following conditions are met:
° Answer was 'Yes' at question '14 [c4]' (Does the unit have a parent organization to which it belongs?)

Please write your answer here:

16 [c6]In which year was the parent organization established? *

Only answer this question if the following conditions are met:
° Answer was 'Yes' at question '14 [c4]' (Does the unit have a parent organization to which it belongs?)

Please write your answer here:

17 [c7]In which year was the drug abuse treatment unit established? *

Please write your answer here:

18 [c8]Would you say that this treatment unit/programme underwent significant operational changes between JUNE 1 2010 AND MAY 31 2011? For example, did significant changes occur with respect to treatment approach, financial support, staff or client composition, etc. *

Please choose the appropriate response for each item:

	Yes	Uncertain	No
Treatment approach	◯	◯	◯
Financial support	◯	◯	◯
Staff composition	◯	◯	◯
Client composition	◯	◯	◯
Number of clients served	◯	◯	◯
Staff to client ratio	◯	◯	◯

Staffing

In this section we will like to know how many staff were engaged during the reference year (June 1st 2010 and May 31 2011) in this treatment unit/programme (or in its parent organization to provide services to this treatment unit/programme) in total and by each staffing category

Indicate the number of staff in each category who attended the TREATNET training.

19 [d1]By categories, how many staff were engaged during the reference year (June 1st 2010 and May 31 2011). Unlike volunteers, Staff is anyone who receives payment regardless of whether they are paid directly by this treatment unit/programme, its parent organization or by other institutions or from public/semi-public sources. If the size of the staff changed during the reference year, give the size that applied to most of the year *

Please enter a number between 1 and 10 for each item:

	Number of full time staff	Number of part time staff	Number of full time volunteers	Number of part time volunteers
Psychiatrists				
Other Physicians				
Psychologists				
Social Workers				
Counsellors (with no formal corresponding qualification)				
Nurses				
Other therapists/ Rehabilitation Specialists/ Trainers/ Educators				
Other Professionals				
Clerks, Administrators, Maintenance personnel				
others				

20 [d2]Are recovering substance abusers employed as staff to provide services in this treatment unit/programme? *

Please choose **only one** of the following:

- ○ Yes
- ○ No

Finances

In this section we would like you to provide information about this treatment unit's/programme's revenues and costs. If possible, base your responses to the questions in this section on income statements, budgets, or other financial documents pertaining specifically to the unit/programme for which you are the person in charge, and not the parent organization or other entity under which you may operate. However, if these financial records are not available, please give your best estimate. Please answer all the questions in this section using data from the last fiscal year. If last fiscal year's data are not available, then use the most recent available fiscal year data. Please be consistent and answer all questions with data from the same fiscal year. Provide answers in Naira.

21 [e1]Was the building space occupied exclusively by this treatment unit/programme in the fiscal year owned, rented or provided free of charge (or at a nominal rent)? *

Please choose **only one** of the following:

- ◯ Owned
- ◯ Rented
- ◯ Provided free of charge by private individual
- ◯ Provided free of charge by the government
- ◯ Building space is shared with some other body free of charge
- ◯ No building space
- ◯ Other [ ]

22 [e2]What are the funding sources of this treatment unit/programme? (PLEASE INDICATE APPROXIMATE PERCENTAGES FOR ALL THAT APPLY AND ADD ANY COMMENT YOU THINK WOULD BE HELPFUL. IF NO COMMENT, TYPE IN 'NA' IN THE RIGHT BOX) *

	percentage(%) (if zero, type in 0)	Any comment? (if no comment, type in "n"or "no")
Federal Government		
State Authorities		
Local Authorities		
International Organization		
Client fees: private income		
Client fees: public insurance		
Client fees: private insurance		
Interest on capital or investments by by the unit		
Donations (charitable)		

Evaluation

To assess available system for evaluating the unit

23 [f1]Does this treatment unit/programme currently undertake or participate in any internal or external evaluation of treatment process or patient outcome? *

Please choose **only one** of the following:

- ◯ Yes
- ◯ No

24 [f2]Who conducts the evaluation? *

Only answer this question if the following conditions are met:
° Answer was 'Yes' at question '23 [f1]' (Does this treatment unit/programme currently undertake or participate in any internal or external evaluation of treatment process or patient outcome?)

Please choose **all** that apply:

- ☐ This treatment unit/programme itself
- ☐ Government institution
- ☐ University or Research institution
- ☐ Independent evaluation consultant
- ☐ Not applicable
- ☐ Other:

25 [f3]Are there reports available on the evaluation data? *

Only answer this question if the following conditions are met:
° Answer was 'Yes' at question '23 [f1]' (Does this treatment unit/programme currently undertake or participate in any internal or external evaluation of treatment process or patient outcome?)

Please choose **only one** of the following:

- ○ Yes
- ○ No
- ○ Not applicable

26 [f4]Give references for the available reports *

Only answer this question if the following conditions are met:
° Answer was 'Yes' at question '23 [f1]' (Does this treatment unit/programme currently undertake or participate in any internal or external evaluation of treatment process or patient outcome?) *and* Answer was 'Yes' at question '25 [f3]' (Are there reports available on the evaluation data?)

Please write your answer here:

RESIDENTIAL TREATMENT UNIT CHARACTERISTICS

This section is for ONLY the treatment unit which deliver drug abuse treatment (and not just housing) where clients may stay overnight. You got to this section because you earlier selected the option that your unit is "specialized residential". If this section does not apply to your unit, kindly navigate backward to de-select "specialized residential"

27 [f1]What type of treatment modality is offered in this treatment unit/programme? (INDICATE ALL THAT APPLY) *

Only answer this question if the following conditions are met:
° Condition: option "specialized residential" was among the options you checked in '12]'

Please choose **all** that apply:

- ☐ Long term drug substitution / maintenance
- ☐ Medication free therapy / long term psychosocial treatment
- ☐ Advice / counselling / support
- ☐ Other: ________

28 [f2]What is the typical planned duration for successful treatment for the majority of clients in this treatment unit/programme? *

Only answer this question if the following conditions are met:
° Condition: option "specialized residential" was among the options you checked in '12]'

Please write your answer here:

____

PLEASE SPECIFY THE UNIT (MONTH OR YEARS)

29 [f3]What is the capacity and intake pattern of your unit? Answer using the table provided below *

Only answer this question if the following conditions are met:
° Condition: option "specialized residential" was among the options you checked in '12]'

	The number
What is your treatment unit's/programme's client capacity? (Number of beds/places for an inpatient service)	
How many clients were admitted/enrolled at this treatment unit/programme, including readmissions, between JUNE 1 2010 AND MAY 31 2011	
How many of the clients were admitted/enrolled more than once between JUNE 1 2010 AND MAY 31 2011? (IF NO READMISSIONS, ENTER 000	
How many new clients (Clients who have not asked for help in this treatment unit before) were admitted/enrolled in this unit/programme between JUNE 1 2010 AND MAY 31 2011?	

ENTER numbers only. If none, enter 0

30 [f4]What percentage of clients currently in treatment and between JUNE 1 2010 AND MAY 31 2011 fall into each special population subgroup listed below *

Only answer this question if the following conditions are met:
° Condition: option "specialized residential" was among the options you checked in '12]'

	Percentage of current admissions	Percentage of ALL admissions JUNE 1 2010 AND MAY 31 2011
Dual diagnosed mental/emotional problems		
Adolescents		
Probationers or parolees		
Females		

31 [f5]written information and treatment plans *

Only answer this question if the following conditions are met:
° Condition: option "specialized residential" was among the options you checked in '12]'

Please choose the appropriate response for each item:

	Yes	Uncertain	No
Does this unit/programme provide written information to clients about the treatment / services offered (e.g., a brochure or other written material)?	○	○	○
Is a written, individual treatment plan usually developed for the clients in this unit/programme	○	○	○
Is the written individual treatment plan based of Addiction Severity Index (ASI)?	○	○	○

32 [f6]What kind of individual treatment/services plan is most common in your unit/programme? *

Only answer this question if the following conditions are met:
° Condition: option "specialized residential" was among the options you checked in '12]'

Please choose **all** that apply:

- ☐ Informal plan
- ☐ Formal written plan - not signed by the client
- ☐ Formal written plan - signed by the client
- ☐ Other: ____________

33 [f7]What types of services are provided to clients, or do clients have access to? *

Only answer this question if the following conditions are met:
° Condition: option "specialized residential" was among the options you checked in '12]'

Please choose the appropriate response for each item:

	No	Yes, On-site	Yes, by referral	Mostly the responsibility of the relatives	Uncertain
Primary Medical Care	○	○	○	○	○

Psychiatric Care	◯	◯	◯	◯	◯
Housing Assistance	◯	◯	◯	◯	◯
School/Academic Training	◯	◯	◯	◯	◯
Vocational Training	◯	◯	◯	◯	◯
Financial Assistance	◯	◯	◯	◯	◯
Job Finding Assistance	◯	◯	◯	◯	◯
Aftercare	◯	◯	◯	◯	◯

34 [f8]Is there an intake or initial assessment (That is appraisal of client's characteristics and needs leading to his acceptance to treatment unit or to referral) service or procedure provided for clients? *

Only answer this question if the following conditions are met:
° Condition: option "specialized residential" was among the options you checked in '12]'

Please choose **only one** of the following:

- ◯ Yes
- ◯ No

35 [f9]Are assessment data kept on paper, in the computer or both? *

Only answer this question if the following conditions are met:
° Condition: option "specialized residential" was among the options you checked in '12]'

Please choose **only one** of the following:

- ◯ Data are kept on paper
- ◯ Data are kept in the computer
- ◯ Data are kept on both paper and in the computer
- ◯ Data are not kept in any record
- ◯ Uncertain

36 [f10]Is the Addiction Severity Index (ASI) routinely administered for intake or initial assessment purposes? *

Only answer this question if the following conditions are met:
° Condition: option "specialized residential" was among the options you checked in '12]'

Please choose **only one** of the following:

- ◯ No
- ◯ ASI is used occasionally
- ◯ ASI is used routinely

37 [f11]Between JUNE 1 2010 AND MAY 31 2011, approximately what percentage of cases graduated

from this treatment unit/programme, was discharged prematurely, or dropped out? *

Only answer this question if the following conditions are met:
° Condition: option "specialized residential" was among the options you checked in '12]'

	Percentage
Treatment completed	
Client dropped out	
Absconded	
Premature discharge due to non-payment of due fees	
Premature discharge due to use of illicit drug within or outside the premises	
Premature discharge due to violent behavior	
Premature discharge due to violation of other rules and regualtions of the unit	

Non-Residential treatment unit characteristics

This section is for only non-residential, low threshold treatment units such as drop-in centres, street agencies, syringe exchange programmes, outreach services, General Practice etc

38 [g1]What is the total overall number of clients who RECIEVED services in this treatment unit between June 2010 and May 2011? (IF UNKNOWN, ENTER 000 IN THE BOX PROVIDED) *

Only answer this question if the following conditions are met:
° Condition: option "specialized residential" was NOT among the options you checked in '12]'

Please write your answer here:

39 [g2]What was the overall number of CONTACTS made by CLIENTS who received services in this treatment unit between June 2010 and May 2011? Note: a client may make multiple contacts. (IF UNKNOWN, ENTER 000) *

Only answer this question if the following conditions are met:
° Condition: option "specialized residential" was NOTamong the options you checked in '12]'

Please write your answer here:

40 [g3]What type of activities/services are organized and delivered by this treatment unit/programme? *

Only answer this question if the following conditions are met:
° Condition: option "specialized residential" was NOT among the options you checked in '12]'

Please choose the appropriate response for each item:

	Yes	Uncertain	No
Advice to drug users	◯	◯	◯
Advice to Non-users	◯	◯	◯
Advice to other service providers	◯	◯	◯
Housing for users	◯	◯	◯
Legal advice/assistance to users	◯	◯	◯
Financial assistance to users	◯	◯	◯
Education	◯	◯	◯
Training	◯	◯	◯
Job finding	◯	◯	◯
Night shelter	◯	◯	◯
Drop in sessions	◯	◯	◯
Relapse Prevention	◯	◯	◯
Prison work	◯	◯	◯
Self help Group work	◯	◯	◯
Counsellor led Group work	◯	◯	◯
On site Syringe exchange	◯	◯	◯
Mobile Syringe exchange	◯	◯	◯
Outreach work	◯	◯	◯
Drug Testing	◯	◯	◯
Medical interventions – primary Health care	◯	◯	◯
Alternative–complementary Therapies	◯	◯	◯

41 [g4]Does this unit/programme provide written information to clients about the treatment/services offered (e.g., a brochure or other written material)? *

Only answer this question if the following conditions are met:
° Condition: option "specialized residential" was NOT among the options you checked in '12]'

Please choose **only one** of the following:

◯ Yes

◯ No

42 [g5] Does this treatment unit/programme keep individual records on clients? *

Only answer this question if the following conditions are met:
° Condition: option "specialized residential" was NOT among the options you checked in '12]'

Please choose **only one** of the following:

◯ Yes

◯ No

43 [g6]Are individual records identifiable? *

Only answer this question if the following conditions are met:

° Condition: option "specialized residential" was NOT among the options you checked in '12]'

Please choose **only one** of the following:

- Yes, by name of client
- Yes, by anonymous identification (code)
- No, only frequency counts of contacts are kept
- Not applicable

44 [g7]Are records kept on paper, in the computer or both? *

Only answer this question if the following conditions are met:
° Condition: option "specialized residential" was NOT among the options you checked in '12]'

Please choose **only one** of the following:

- Records are kept on paper
- Records are kept in the computer
- Records are kept on both paper and in the computer
- Not apllicable
