# Supplementary material for: A descriptive survey of types, spread and characteristics of substance abuse treatment centers in Nigeria
Source: Subst Abuse Treat Prev Policy. 2011 Sep 18;6:25. doi: 10.1186/1747-597X-6-25 (PMC3182902; doi:10.1186/1747-597X-6-25)
Supplement: Additional file 2 — Substance abuse treatment unit screening form. The form distinguishes between generic treatment units and substance abuse treatment specific units. [file 1747-597X-6-25-S2.PDF]

Substance abuse treatment unit screening form

Dear colleague, thank you for participating in the TREATNET training. Kindly spare 3 to 5 minutes to fill this form to enable us build the directory of our treatment units. The directory ***is not really about the parent hospital or institution where your unit is based***. We know that it is wrong to assume that all TREATNET trainees from the same hospital work in the same treatment unit. So respond only as it relates to the unit where you work.

Your surname	
Your other names	
Your phone number	
Your emails	

Which of the following **BEST** describes the setting of the treatment unit where you work? (SELECT ONLY ONE OPTION)

A[ ]	It is a unit or ward which is OFFICIALLY dedicated for drug dependence treatment in the psychiatric department of a UNIVERSITY TEACHING hospital
B[ ]	It is a unit or ward which is NOT OFFICIALLY dedicated for drug dependence treatment in the psychiatric department of a UNIVERSITY TEACHING hospital.
C[ ]	It is a unit or ward which is OFFICIALLY dedicated for drug dependence treatment in the psychiatric department of a GENERAL hospital
D[ ]	It is a unit or ward which is NOT OFFICIALLY dedicated for drug dependence treatment in the psychiatric department of a GENERAL hospital
E[ ]	It is a unit or ward which is OFFICIALLY dedicated for drug dependence treatment in a SPECIALIST PSYCHIATRIC hospital
F[ ]	It is a unit or ward which is NOT OFFICIALLY dedicated for drug dependence treatment in a SPECIALIST PSYCHIATRIC hospital
G[ ]	It is a general medical practice
H[ ]	It is a primary health care setting
I[ ]	It is a stand-alone drug dependence treatment unit (not part of a parent hospital)
J[ ]	It is a unit based in prison or any other law enforcement institution.
K[ ]	It is primarily a HIV/AID treatment unit.
L[ ]	It is a unit in the administrative arm of government, e.g. ministry of health
M[ ]	It is a social group promoting prevention and treatment of drug dependence<br>Others (specify)____

The name of your unit as it is OFFICIALLY called is?

Name of the parent hospital (If applicable) is?

State where located

Your unit is based in which of these sectors?

[ ]	Federal government
[ ]	State government
[ ]	Private sector
[ ]	Non-governmental organization

Are you in the position to be TREATNET contact person for your unit? A TREATNET contact person in your unit will later give further details about your unit for the purpose of needs assessment of substance abuse treatment centres in Nigeria. The person should be a Treatnet trainee and have good working relationship with the head of the unit if he is not the head.

[ ]	YES, I am in the position to be the TREATNET contact person for my unit.
[ ]	NO, I will like to suggest someone else in my unit to the TREATNET contact person for my unit.
